# Symptom-specific links between internalizing problems and functional connectivity in adolescents: a network analysis

**DOI:** 10.1007/s00787-026-02989-6

**Published:** 2026-02-25

**Authors:** Valerie Karl, Ludvig D. Bjørndal, Eira R. Aksnes, Irene J. E. Teulings, Niamh MacSweeney, Dani Beck, Lars T. Westlye, Omid V. Ebrahimi, Christian K. Tamnes

**Affiliations:** 1 PROMENTA Research Center, Department of Psychology, University of Oslo, Blindern, PO Box 1094, 0317 Oslo, Norway; 2 Psychiatric Genetic Epidemiology Group, Research Department, Lovisenberg Diaconal Hospital, Oslo, Norway; 3 Division of Mental Health and Substance Abuse, Diakonhjemmet Hospital, Oslo, Norway; 4 Department of Psychology, University of Oslo, Oslo, Norway; 5 K.G. Jebsen Centre for Neurodevelopmental Disorders, University of Oslo, Oslo, Norway; 6 Center for Precision Psychiatry, Division of Mental Health and Addiction, Oslo University Hospital, Oslo, Norway; 7 Department of Experimental Psychology, University of Oxford, Oxford, UK; 8 Department of Psychiatry, University of Oxford, Oxford, UK

**Keywords:** Network analysis, Functional connectivity, Internalizing problems, Youth, ABCD, Symptom networks

## Abstract

Previous fMRI studies have documented links between internalizing problems in youth and brain functional connectivity of the default (DN), frontoparietal (FP), and salience (SA) networks. Characterized by a large symptom heterogeneity and comorbidity, it remains elusive how individual internalizing symptoms relate to DN, FP, and SA connectivity. Leveraging a large population-based sample of adolescents (*N* = 2426; mean age = 14.1 years) and an integrated network modelling approach, we identified symptom-specific associations between internalizing problems and functional connectivity and explored sex and timing-specific differences in these links. Our findings revealed small negative associations between self-reported feelings of worthlessness and guilt and DN within-network connectivity and positive associations between fearfulness and FP within-network connectivity. Moreover, sadness and fearfulness were positively associated with DN-SA between-network connectivity. Exploratory analyses revealed no significant sex differences but indicated that DN within-network connectivity around age 10 was negatively associated with self-reported worthlessness at age 14. Our findings show symptom-specific associations between internalizing problems and brain functional circuitry in youth and highlight the complex interplay of symptoms and brain networks.

**Graphical Abstract:**

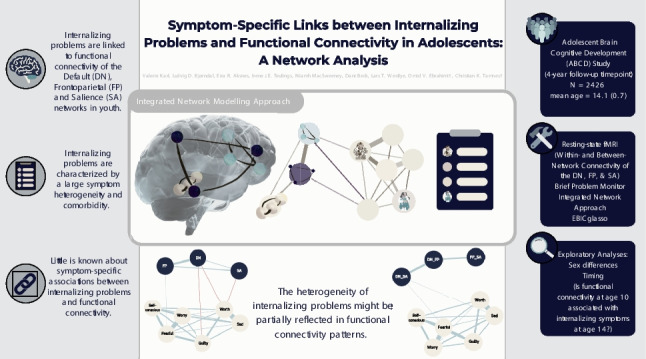

**Supplementary Information:**

The online version contains supplementary material available at 10.1007/s00787-026-02989-6.

## Introduction

Internalizing problems, including depression and anxiety disorders, often emerge during adolescence and are increasingly prevalent among female youth [1–3]. Both depression and anxiety have been linked to disrupted brain functional connectivity of the default (DN), frontoparietal (FP), and salience (SA) network [4, 5]. Internalizing problems are characterized by high symptom heterogeneity and comorbidity, yet how this relates to functional connectivity remains poorly understood [6, 7]. By leveraging a large adolescent neuroimaging dataset and network modelling, we aimed to characterize symptom-specific links between internalizing problems and functional circuitry in the developing brain.

Categorical classifications of mental disorders are based on observed and/or reported patterns of symptoms and behaviors, yet they neglect the high symptom heterogeneity among individuals diagnosed with the same disorder (e.g. major depressive disorder (MDD)) nor consider varying symptom profiles across age groups, such as in adolescents and adults [7–9]. Alternative approaches to conceptualizing mental disorders, such as symptom networks, allow an investigation of associations between mental disorders and their neural correlates on more granular phenotypic resolutions [10].

From a network perspective, mental disorders are conceptualized as systems of interacting symptoms and do not have a single underlying common cause [11]. Accordingly, mental disorders are understood to emerge when symptoms (e.g., sad mood) are activated (e.g., by an external event, such as the loss of a partner or disrupted sleep) and directly influence neighboring symptoms (e.g., sad mood increases feelings of worthlessness). Symptom interrelationships and feedback loops can thereby ultimately lead to stable and “self-sustaining” disordered states. Naturally occurring interactions between symptoms potentially stem from underlying biopsychosocial processes [11]. Nevertheless, it remains unclear whether symptoms interact and co-occur due to shared neural underpinnings [12].

Alongside clinical psychology, network science has profoundly shaped the field of neuroscience [13, 14]. Functional brain networks are communities of brain regions with correlating neural activity [15, 16] that can be examined with functional magnetic resonance imaging (fMRI). Importantly, psychopathology has been associated with atypical connectivity patterns of three large-scale functional brain networks: the DN, FP, and SA [4]. The DN is implicated in self-referential thoughts, auto-biographical memory, and perspective taking and enhanced connectivity of its regions has been linked to depression in adolescents [5, 17] and adults [18]. The FP (also called central executive network) is involved in cognitive control and its within-connectivity is positively associated with anxiety symptoms in youth [5]. The SA detects, processes, and integrates salient information and can switch between recruiting either the DN or FP [19, 20]. Adolescent depression has been associated with SA expansion (i.e., an encroachment of other regions) in children before depression onset [21], while cross-sectional studies indicate that anxious behavior and thoughts are related to weakened SA within-connectivity in adolescents and adults [22–24]. During adolescent development, connectivity of regions within a network typically strengthens while connectivity between networks weakens [25], yet higher connectivity between DN and both FP and SA has been consistently linked to internalizing problems [5, 26, 27].

Network models have shaped our understanding of mental disorders in both clinical psychology [11, 28] and neuroscience [29, 30], but their methodological approaches vary considerably (e.g., node definition, measurement dimension). In the field of clinical psychology, network analysis comprises a set of methods which are well-suited to examine symptom interrelationships and their associations with different risk factors [31]. In neuroscience, network approaches can yield information on structural and functional connections of brain regions and point towards potentially dysfunctional interplay between regions [4, 12, 32]. However, a framework for integrating symptom and brain networks was recently introduced [12], paving a new path towards the study of symptom-specific associations between internalizing problems and brain networks.

Symptom network analyses have identified depressed mood and worry as the most central (i.e., the most strongly connected) symptoms in internalizing symptom networks [33] and found to predict future symptoms in adolescents [34, 35]. Few studies have examined symptom-specific links between internalizing problems and brain imaging metrics. Analyses of fMRI data have shown links between symptom-specific fluctuations of anhedonia and SA connectivity in adults with depression [36], as well as associations between negative thoughts in late adolescent-onset depression and dynamic functional connectivity transitions in the DN [37]. Brain-behavior analyses applying integrated network modelling [12] have provided initial evidence for symptom-specific links between depressive symptoms and brain structure [38, 39]. Furthermore, incidence rates, symptom network characteristics, and associations between psychopathology and functional connectivity vary notably across male and female youth [1, 2, 40–42]. Associations between symptom networks of internalizing problems and adolescents’ functional brain networks have not been examined, nor has it been explored whether these associations vary by sex.

This study aimed to investigate the complex interplay of internalizing symptoms and brain network connectivity in youth by means of network modelling. Using an integrated network approach [12], we examined symptom-specific links between self-reported internalizing symptoms and within and between functional connectivity measures derived from resting-state fMRI data in a well-powered youth sample (*N* = 2426; mean age = 14.1 years). Based on previous findings reviewed above, we expected to find positive associations between internalizing symptoms and between-connectivity measures. Overall, based on findings showing symptom-specific links to functional connectivity [36, 37], we expected the links between internalizing problems and atypical network organization to be symptom-specific rather than uniform across all symptoms. For exploratory purposes, we additionally tested if functional connectivity at age 10 was associated with later internalizing symptoms, and whether networks differed between males and females.

## Methods

### Sample

The final sample consisted of 2426 adolescents (mean age 14.1, SD = 0.7, female = 47.0%) from the 4-year follow-up timepoint of the Adolescent Brain Cognitive Development (ABCD) Study (release 5.1; 10.15154/z563-zd24). This large-scale multi-site study recruited ~ 11.800 participants at the age of 9–10 years between 2016 and 2018, with plans to follow them for 10 years. The ABCD Study was approved by the Institutional Review Board at the University of California San Diego [43]. Parents or guardians provided written consent, while the child provided written assent. Details on the data collection procedures and study protocols can be found elsewhere [44–46]. Based on the current study’s focus on internalizing problems, which are more prevalent during mid-adolescence than early adolescence [1], our main analyses included data from the 4-year follow-up timepoint, which was available for 2744 participants with either female or male sex assigned at birth. We randomly picked one sibling per family (seed set to 13) to control for potential confounds due to relatedness of the participants in the sample. For exploratory purposes, we utilized resting-state fMRI data collected at the ABCD Study baseline timepoint, which was available for 1997 adolescents (mean age = 10.0, SD = 0.6) of the sample included in the main analyses. An overview of the data used in the analyses is illustrated in Supplementary Figure S1.

### Brief problem monitor

Youth internalizing problems were assessed with the Brief Problem Monitor (BPM, [47]). As part of the Achenbach System of Empirically Based Assessment (ASEBA), [47, 48] this 19-item self-report questionnaire is designed to measure 6- to 18-year olds’ mental health within the past week. Items were rated as “not true (0)”, “somewhat or sometimes true (1)”, or “very true or often true (2)” and can then be summarized in internalizing, externalizing, attention, and total problems scales, with satisfactory to high internal consistency (Cronbach’s *α*: 0.79—0.91) [49]. In this study, we used the 4-year follow-up ratings on the 6 items that make up the internalizing scale: “I feel worthless or inferior”, “I am too fearful or anxious”, “I feel too guilty”, “I am self-conscious or easily embarrassed”, “I am unhappy, sad, or depressed”, and “I worry a lot”.

### Imaging acquisition

MRI data was collected on 32 different 3-T scanners (either Siemens Prisma, General Electric 750, or Phillips) across 22 study sites in the USA. Scanning parameters were harmonized across sites. Resting-state fMRI data was acquired with multiband echo-planar imaging (TR = 800 ms, TE = 30 ms, flip angle = 52°, and FOV = 216 mm^2^) during four five-minute scanning sessions. Participants were instructed to keep their eyes fixated on a crosshair during these scans. Further details on imaging protocols can be found in Casey et al. [44].

### MRI preprocessing procedure

MRI processing was conducted by the ABCD Study Data Analysis and Informatics Core team and involved a standardized ABCD Study pipeline (see [50], for details). In short, the pipeline included removal of initial volumes, normalization and detrending of the timeseries, linear regression to remove trends and signals of motion, white-matter, ventricles, whole brain, and first derivatives [51, 52]. Frames with a displacement over 0.3 mm were excluded from the regression. Lastly, time courses were temporal band-pass filtered between 0.009 and 0.08 Hz [53] before being mapped onto individual cortical surfaces and filtered for motion associated with respiration [51, 54].

Functional connectivity measures were derived using a seed-based, correlational approach [55] for cortical surface based analyses [56]. Average time courses were calculated for 333 cortical surface-based ROIs using a functionally-defined parcellation based on resting-state functional connectivity patterns [15] and 19 subcortical ROIs [57]. Next, Pearson’s product moment correlation coefficients between the average time courses of each ROI-pairing were calculated and normalized with Fisher’s r-to-z transformation. ROIs were then grouped into pre-defined Gordon networks (e.g., DN, auditory network, dorsal attention network). Within- and between network correlation strength was then extracted by calculating the mean Fisher-transformed correlation coefficient of the respective ROI-pairings. These coefficients served as indices of functional connectivity strength within and between networks in this study. As atypical connectivity patterns of DN, FP, and SA have been linked to psychopathology [4], we focused on within- and between-connectivity measures of these networks (Figure S2).

### Statistical analyses

All statistical analyses were conducted in R (version 4.3.2; [58]). Resting-state fMRI connectivity variables were adjusted for scanner effects using the R package *neuroComBat* (version 1.0.13) [59], with all internalizing symptom items, age, and sex included into the Combat model. To adjust for age and sex effects, we residualized the effects of age and sex on both the symptom and functional connectivity variables prior to the statistical analyses (see Supplementary Figures S3 and S4 for results).

#### Network estimation

In our main analysis focusing on identifying symptom-specific links between internalizing problems and brain functional connectivity, we estimated Gaussian Graphical Models (GGMs) using the R package *bootnet* (version 1.6) [60], and utilized the built-in *cor_auto* function, which selects appropriate correlation coefficients based on the measurement level of each variable. These undirected networks included internalizing symptoms (*n* = 6) and functional connectivity variables (*n* = 3) as nodes and their conditional associations as edges. The resulting edge weights thus represent pairwise partial correlations, revealing the unique association between two nodes after the effects of all other variables (nodes) have been taken into account. Separate networks were estimated for measures of symptoms and within-network connectivity and for symptoms and between-network connectivity. All items were scaled prior to network estimation. We used the graphical least absolute shrinkage and selection operator (GLASSO), which penalizes the parameter estimates to reduce the likelihood of false positives [61]. This regularization technique yields a sparse network that includes the most important remaining edges. In a final step, the Extended Bayesian Information Criterion (EBIC) for model selection determined which of the estimated models yielded by GLASSO showed the best model fit [60, 62]. All networks were visualized with the Fruchterman-Reingold algorithm using the R package *qgraph* (version 1.9.8) [63].

#### Exploratory analyses

First, to test whether functional brain network patterns at 10 years old predicted specific internalizing symptoms in participants four years later, we repeated the main analyses with baseline functional connectivity measures from 1997 adolescents (mean age = 10.0, SD = 0.6) of the 2426 participants included in the main analysis. We here repeated the steps described above (adjusting the data for scanner-site effects and residualizing the effects of age at baseline and sex), before estimating networks with functional brain connectivity measures from baseline and internalizing symptoms from the 4-year follow-up timepoint as nodes. In addition, we repeated these analyses while controlling for symptom levels at the 6-month follow-up (the earliest timepoint at which the BPM was administered) to examine whether functional connectivity at age 10 was associated with changes in internalizing symptoms during early adolescence. Second, due to the well-established sex differences in internalizing problems and association with rsFC [2, 41], we conducted exploratory analyses testing if symptom-brain networks significantly differed between males and females. Statistical comparisons were conducted using the (individual) network invariance test (NIT) [64] implemented in the R package *psychonetrics* (version 0.13) [65], which allowed us to test differences between two networks (here: networks for males and females). To this end, we used symptom and brain data (measured at age 14 and harmonized across scanning sites) and only residualized age effects from the data. We estimated a heterogeneous model, in which all edges were freely estimated (i.e., edges can be different between females and males) as well as a homogeneous model with edge weights constrained to be equal across sexes (testing a model where there are no differences between females and males). These two models were then compared with a lower resulting Akaike information criterion (AIC) indicating which model fit the data better [64, 66]. Third, to quantify the overall association between internalizing problems and connectivity measures, we conducted linear models with the connectivity measures as the dependent variable, the BPM internalizing problem sum score, age, and sex as fixed effects. Here, we calculated the sum of all six items to represent a general internalizing problem scale [47]. Fourth, we conducted a unified analysis with internalizing symptoms and both within- and between-connectivity measures to explore interactive patters between within- and between-network connectivity measures in relation to symptoms.

#### Network stability analysis and sample variation

The relative importance of nodes was assessed with node strength estimates, which we computed by summing up all absolute edge weights connecting the node to other nodes. To test the robustness and accuracy of the estimated edge weights against sampling variation we applied non-parametric bootstrapping with 1000 iterations and estimated 95% confidence intervals (CIs) [60]. Next, we assessed the stability of the networks’ centrality measures by means of case-dropping bootstrapping analyses. Centrality measures of the full sample were estimated and subsequently correlated with re-estimated centrality measures that were computed based on subsets of the data. This procedure was repeated 1000 times, yielding the correlation stability (CS) coefficient. The CS coefficient indicates the maximum proportion of participants that can be dropped from the sample, so that the correlation between the original sample centrality measures and the newly estimated subset centrality measures is (with a 95% probability) at least 0.7. This CS-coefficient should be minimum 0.5 [60].

## Results

**Fig. 1:**
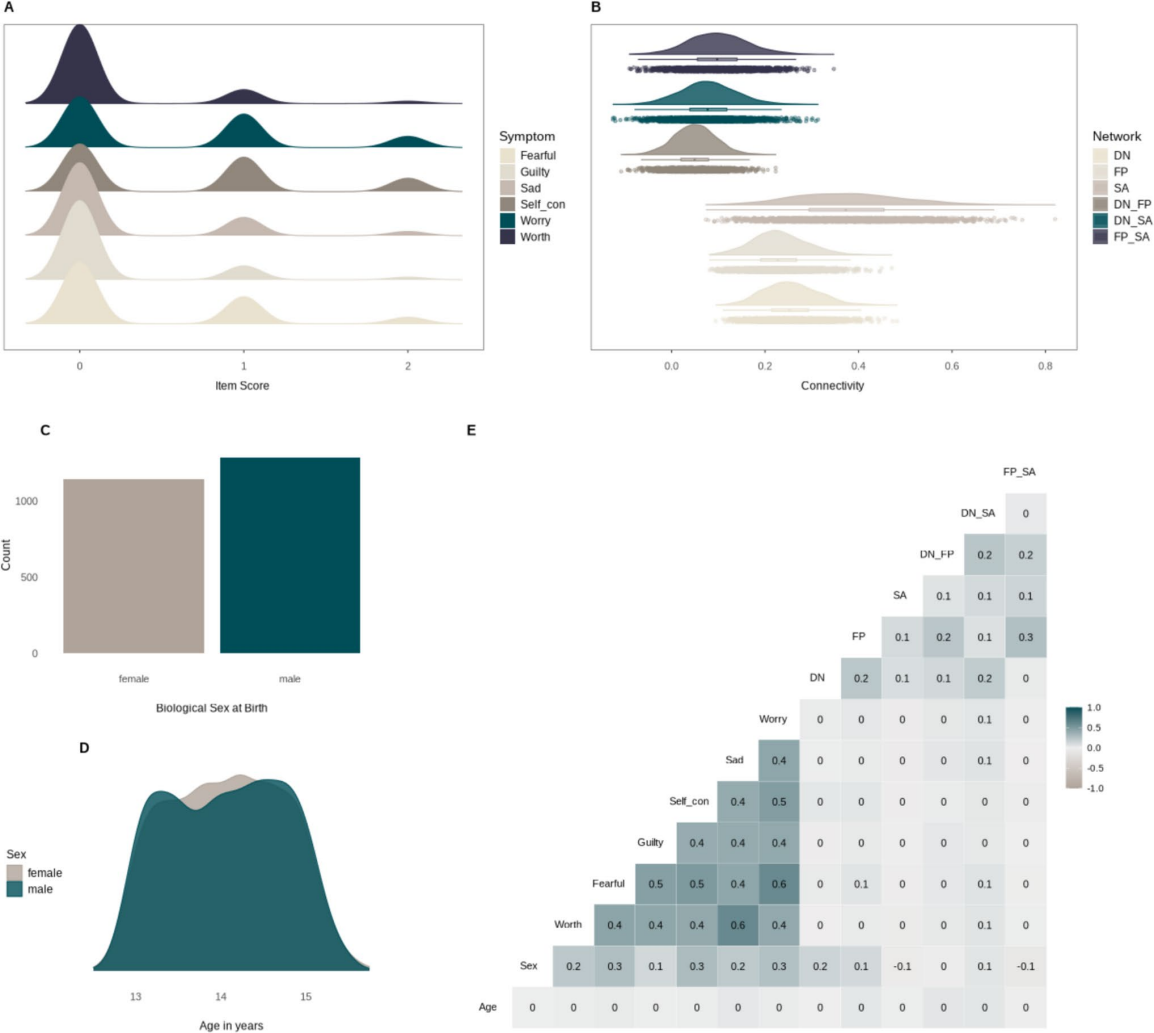
Overview of sample characteristics: demographics, internalizing problems, and functional connectivity. **A**: Distribution of symptom ratings. **B**: Average within- and between-network connectivity at age 14. **C** and **D**: Distribution of sex, and age by sex. **E**: Pearson’s correlation coefficients between symptom ratings, functional connectivity measures, age, and sex

Sample characteristics are summarized in Table 1 and Fig. 1. Network analysis results are reported in accordance with guidelines for cross-sectional network studies [67].

**Table 1:** Sample characteristics: age, sex, and ethnicity

Age		
	Mean	14.1
	*SD*	0.7
	Mean_baseline_	10.0
	SD_baseline_	0.6
Biological sex		
	Male	53.0%
	Female	47.0%
Ethnicity		
	Asian	2.4%
	Black	10.2%
	Hispanic	20.4%
	White	56.6%
	Other	10.4%

Demographics of the main sample including information on youth age, sex, and ethnicity. N_Total_ = 2426; N_baseline_ = 1997

### Network analysis of internalizing symptoms and within-network connectivity

**Fig. 2:**
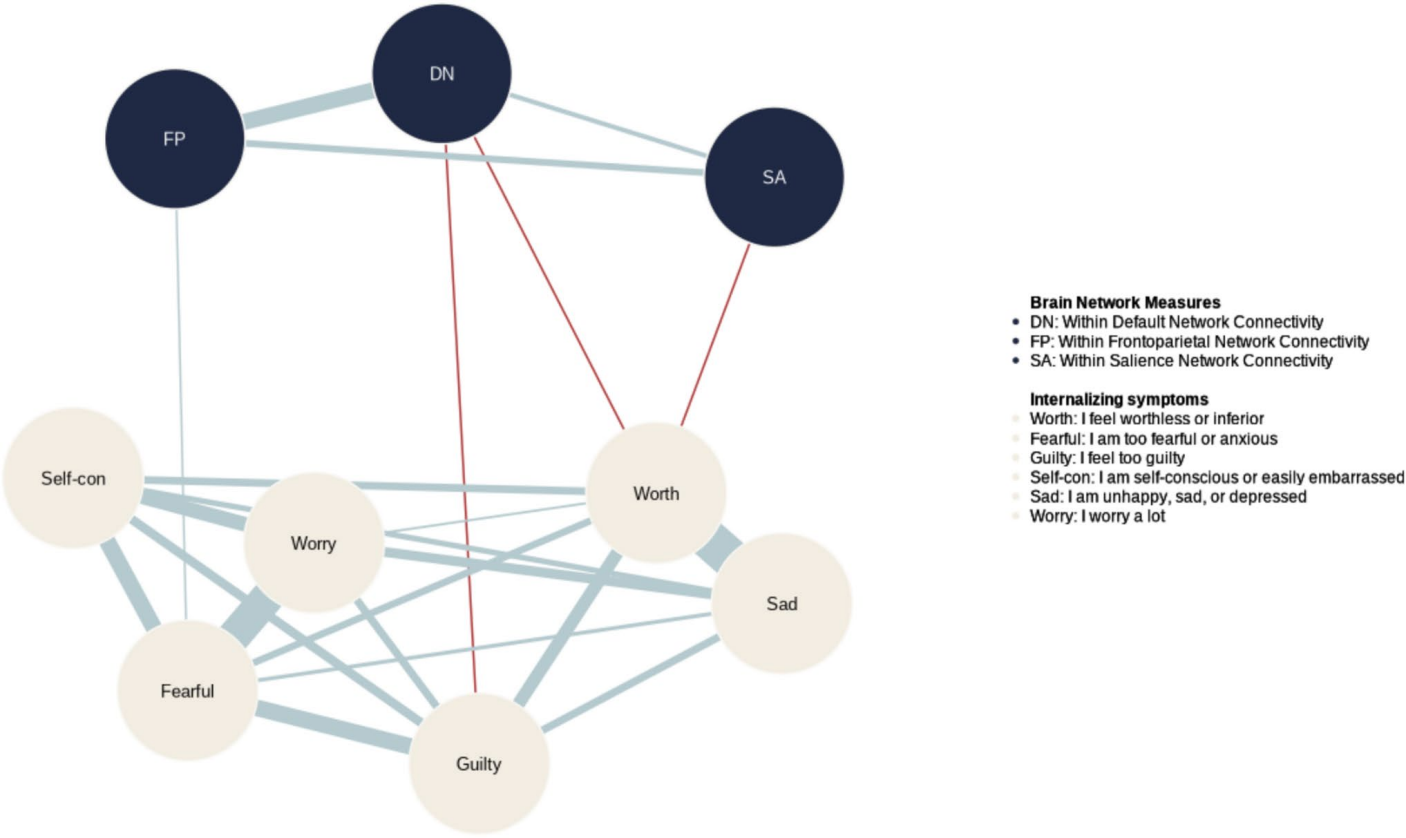
Network plot of internalizing symptoms and within-network connectivity. Network graph depicting conditional associations between internalizing symptoms and network within-connectivity measured at age 14. Blue edges indicate positive associations, and red edges represent negative associations. To enhance the visibility of cross-domain edges, we set the cut parameter to 0.04, thereby adjusting the scaling of edge weights

Within-domain associations were higher than cross-domain associations, with the strongest conditional relationships between self-reported worthlessness and sadness (*r* = 0.438) and worry and fearfulness (*r* = 0.368) and for neural measures between within-connectivity of the DN and the FP (*r* = 0.191). Linking symptoms with within-network connectivity measures, we found small negative conditional associations between self-reported guilt (*r* = −0.017) and within-connectivity of the DN and between worthlessness and within-network connectivity of the DN (*r* = −0.012) and SA (*r* = −0.011). Moreover, self-reported fearfulness was positively associated with FP within-network connectivity (*r* = 0.009). A visualization of the estimated regularized network for internalizing symptoms and within-connectivity measures can be found in Fig. 2 and edge weights are presented in Supplementary Table S1.

### Network analysis of internalizing symptoms and between-network connectivity

**Fig. 3:**
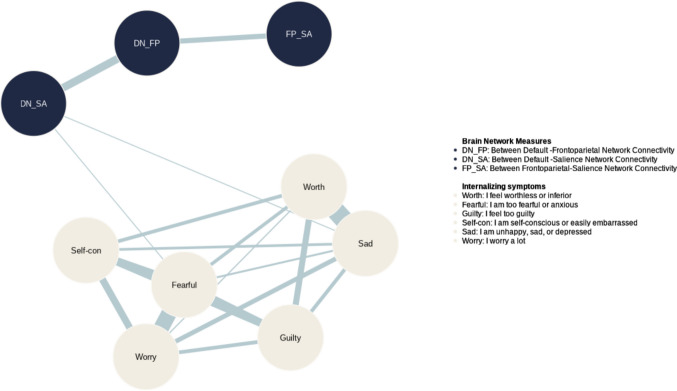
Network plot of internalizing symptoms and between-network connectivity. Network plot depicting conditional associations between internalizing symptoms and network between-connectivity at age 14. Blue edges indicate positive associations. To improve the visibility of cross-domain edges, we set the cut parameter to 0.04

For networks with between-network connectivity measures the strongest associations remained between worthlessness and sadness (*r* = 0.423) and between fearfulness and worry (*r* = 0.356) for symptom associations and between the nodes representing DN-SA and DN-FP connectivity (*r* = 0.189) for neural measures. We found small positive edges linking fearfulness and sadness to DN-SA connectivity (*r* = 0.003–0.004). The network plot with internalizing symptom and between-connectivity measures is displayed in Fig. 3 and edge weights are reported in Supplementary Table S2.

### Node strength

Node strength is represented in radar plots in Supplementary Figure S5 and indicates the relative importance of fearfulness (total sum edge weight = 0.89–0.92) in both networks. The node with smallest strength was FP (total sum edge weight = 0.29) in the within and DN-FP (total sum edge weight = 0.32) in the between network.

### Stability and accuracy

**Fig. 4:**
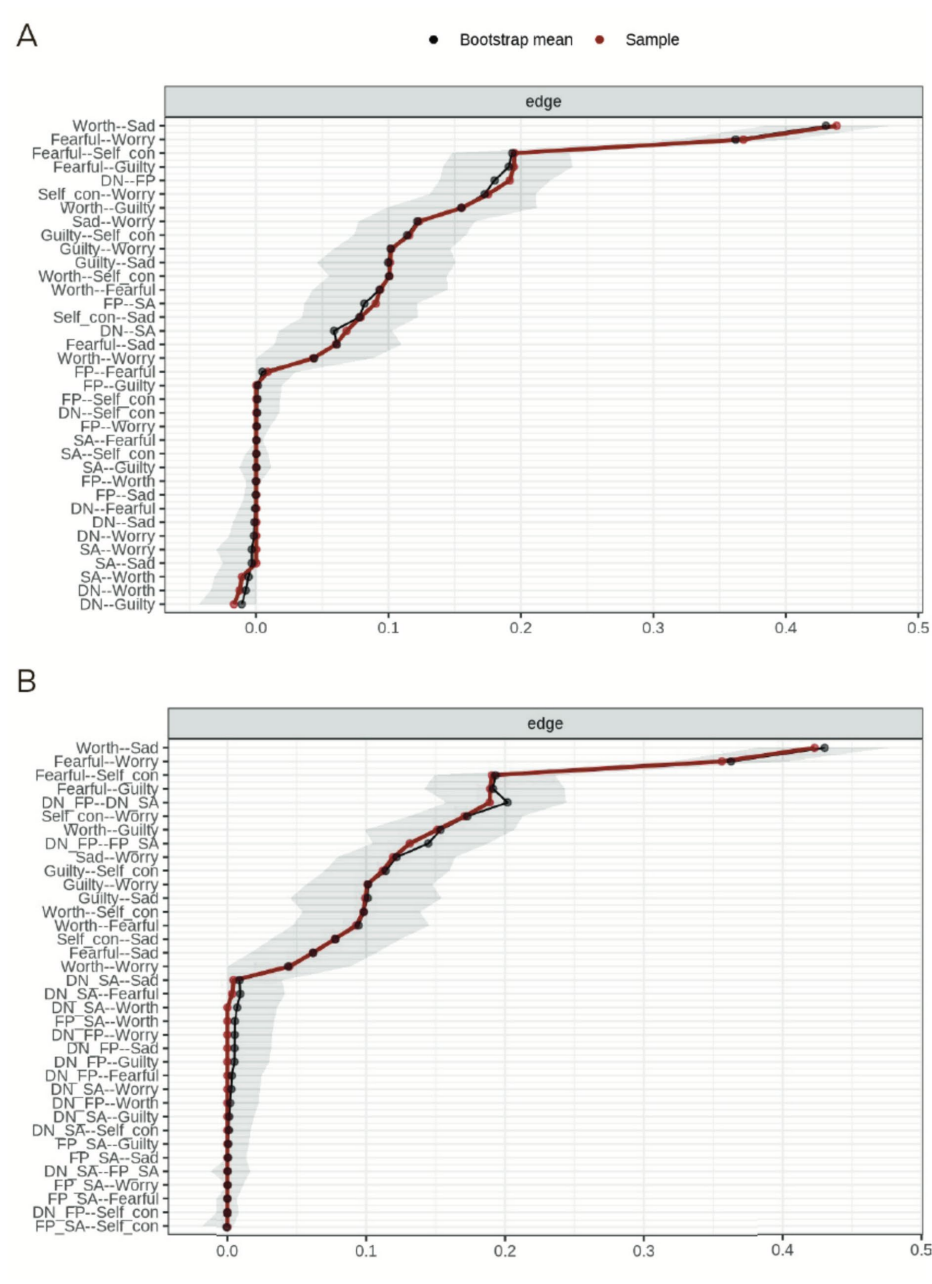
Estimated bootstrap intervals. **A**: Within-network Connectivity and Internalizing Symptoms at age 14. **B**: Between-network Connectivity and Internalizing Symptoms at age 14

Detailed results of the accuracy and stability analyses are reported in Fig. 4. Bootstrapping yielded narrow 95% CIs, indicating high accuracy of the estimated edge weights [60]. For the cross-domain edges, however, the bootstrapped CIs included zero (CI_DN-Guilty_[−0.04—0.01], CI_DN-Worth_[−0.03—0.01], CI_FP-Fearful_[−0.01—0.03], CI_SA-Worth_[−0.03—0.01], CI_DN_SA-Fearful_[−0.02—0.03], CI_DN_SA-Sad_[−0.02—0.03]. As noted by Epskamp et al. [60], bootstrapped CIs should not be interpreted as significance tests in LASSO-regularized networks, but rather as an indication of the limited precision of edge weight estimation [60]. Furthermore, CS-coefficients of 0.75 for both within- and between-network connectivity measures provided evidence of high stability in the obtained centrality measures.

### Exploratory analyses

**Fig. 5:**
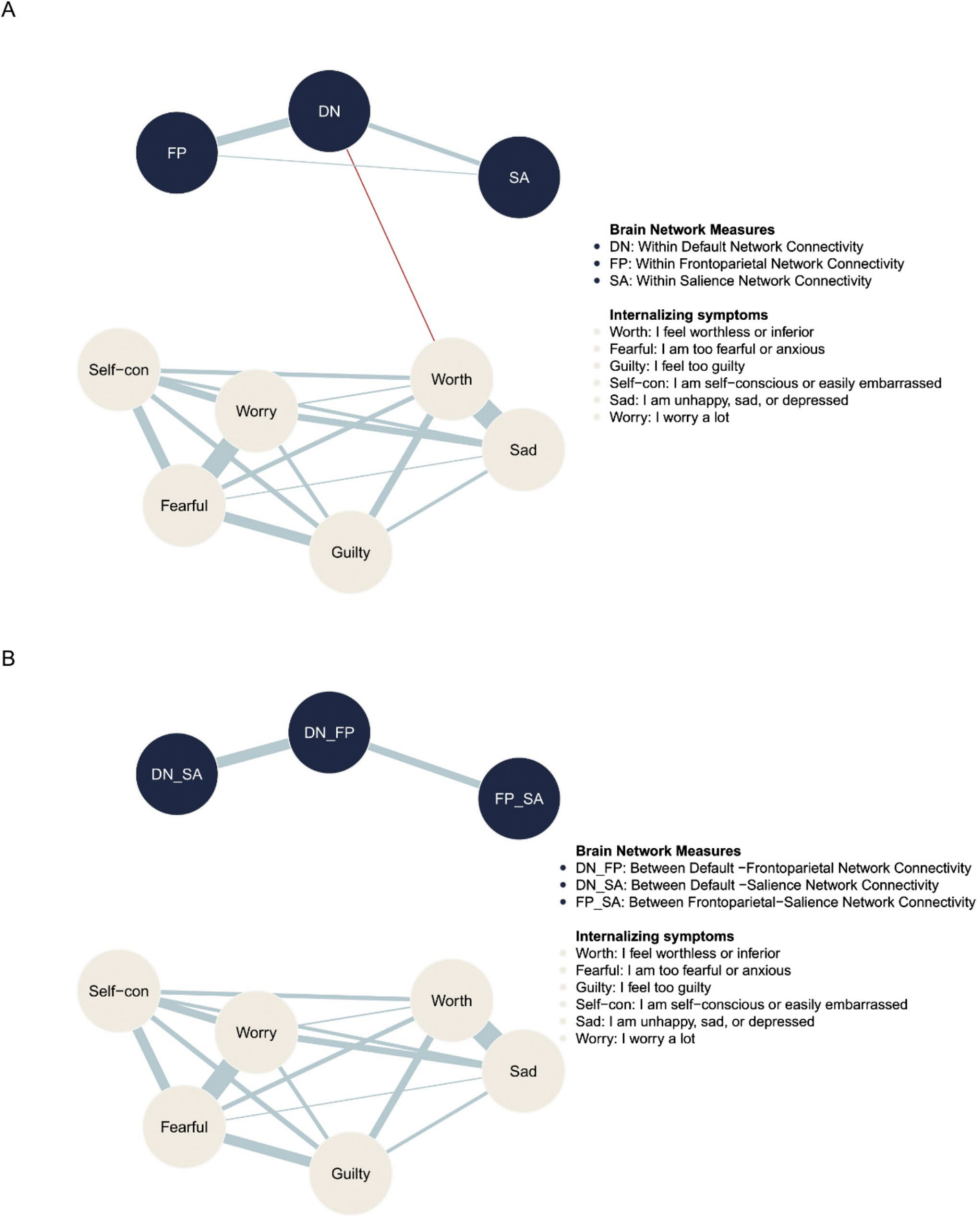
Network plots of within- and between-network connectivity at age 10 and internalizing symptoms at age 14. Network plots depicting conditional associations between **A** within-network and **B** between-network functional connectivity at age 10 and internalizing symptoms at age 14. Blue edges indicate positive associations, and red edges represent negative associations. The cut parameter was set to 0.04 to highlight cross-domain edges

**Fig. 6:**
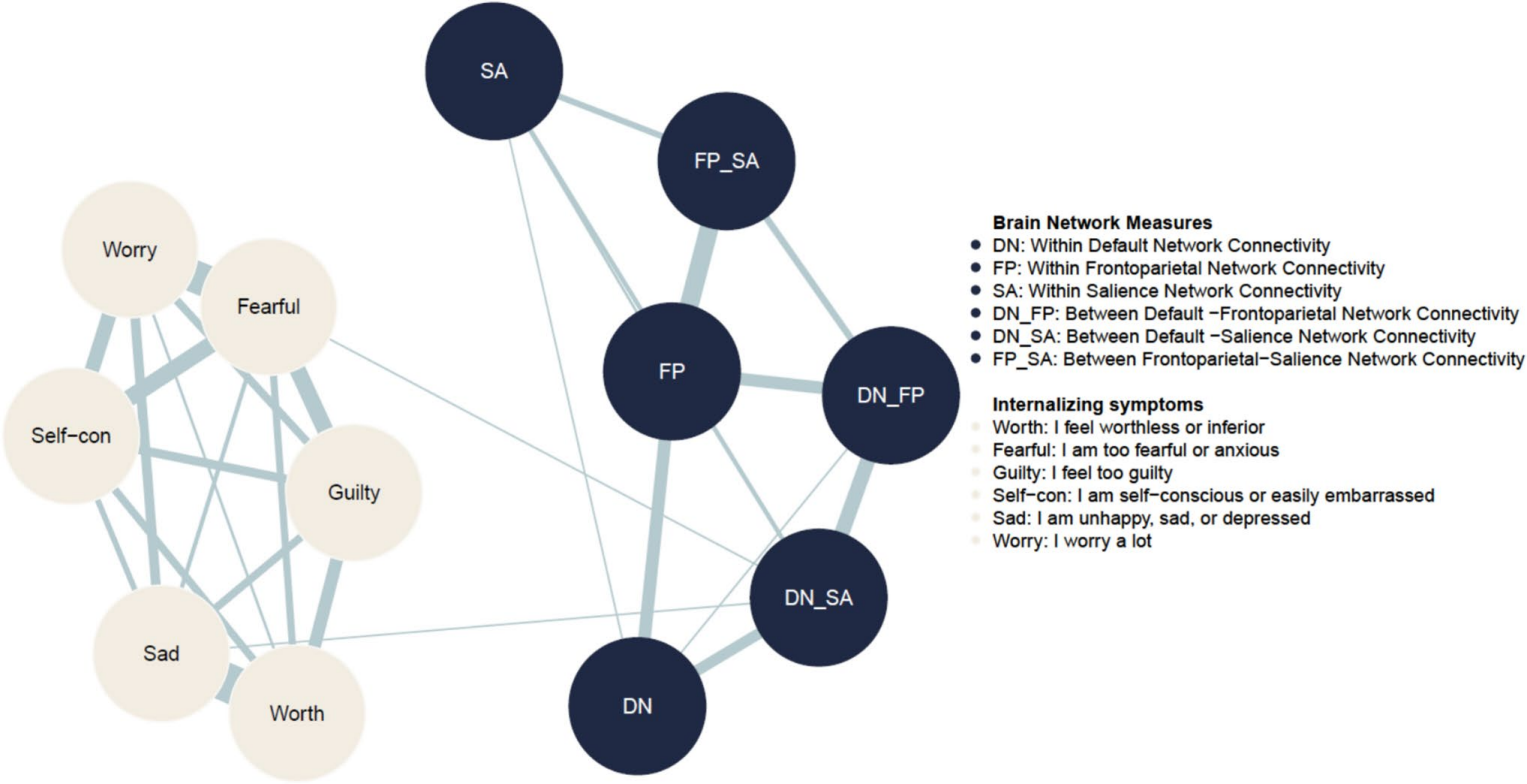
Network plot of internalizing symptoms and both within-network and between-network connectivity measures. Network plot depicting conditional associations between internalizing symptoms and both within-network and between-network connectivity at age 14. Blue edges indicate positive associations. To improve the visibility of cross-domain edges, we set the cut parameter to 0.04

First, we tested if functional connectivity at age 10 was related to internalizing symptoms at age 14. Here, we found a negative association between within-DN connectivity measured at age 10 and feelings of worthlessness reported at age 14 (*r* = −0.010). These networks are displayed in Fig. 5A (within-connectivity measures) and 5B (between-connectivity measures) and edge weights are reported in Supplementary Tables (S3 and S4). The cross-domain association between feelings of worthlessness and within-DN connectivity disappeared when controlling for self-reported feelings of worthlessness at the 6-month follow-up. Second, we examined whether symptom-specific associations between internalizing problems and functional connectivity at age 14 differed between males and females. The analyses did not indicate a significant difference between networks for males and females for neither networks estimated with within-network connectivity (AIC_equal_ = 56103.85 < AIC_different_ = 56121.38), nor between-network connectivity (AIC_equal_ = 56120.98 < AIC_different_ = 56133.02) measures. Third, to test for an overall link between total internalizing problems and functional connectivity, we conducted linear models that showed significant positive associations between the internalizing problem sum score and DN-SA between-connectivity (*β* = 0.05, *p* = 0.007, *p*_*FDR-corrected*_ = 0.042) and DN-FP between-connectivity (*β* = 0.05, *p* = 0.025, *p*_*FDR-corrected*_ = 0.074). Fourth, we integrated internalizing symptoms and both within-network and between-network connectivity in a unified network. While this network did not reveal cross-domain edges between internalizing symptoms and within-network connectivity measures, unique associations between self-reported fearfulness (*r* = 0.002, CI = [−0.02—0.2]) and sadness (*r* = 0.003, CI = [−0.02—0.2]) and DN-SA between-network connectivity were also observed in the unified network (see Fig. 6).

## Discussion

To probe symptom-specific links between internalizing problems and brain functional connectivity in adolescents, we used an integrated network modelling approach [12], combining symptom scores and measures of within- and between-network connectivity measures of the DN, FP, and SA. We identified negative conditional associations between self-reported feelings of worthlessness and guilt and DN within-network connectivity. Worthlessness additionally showed a negative association with SA within-network connectivity and fearfulness was positively linked to FP within-network connectivity. Moreover, self-reported sadness and fearfulness exhibited positive associations with DN-SA between-network connectivity. Our analyses revealed no significant sex differences in brain-symptom networks. Lastly, we found that 14-year old’s self-reported worthlessness was associated with DN within-network connectivity at age 10. Overall, our findings unravel granular associations between individual internalizing symptoms and functional brain networks in adolescence.

Self-reported feelings of guilt and worthlessness were uniquely associated with DN within-network connectivity and might reflect the DN’s involvement in self-referential and self-generated thoughts [68–70]. In contrast to the negative association between DN within-network connectivity and depressive symptoms in this study, increased connectivity between regions of the DN has previously been observed in both youth [5, 71] and adult cohorts [18, 72] with depressive problems. Furthermore, Marchitelli et al. [37] reported symptom-specific associations between negative thoughts and DN connectivity changes in late adolescent-onset MDD. Both overall and symptom-specific (sadness and fearfulness) measures of internalizing problems exhibited positive associations with DN-SA connectivity. Altered connectivity between DN and SA has been linked to an attentional bias towards negative information and might contribute to negative self-referential processing and maladaptive rumination found in depression [73–77]. However, it remains unclear whether functional connectivity within the DN or between the DN and other networks is more relevant for internalizing problems. The disappearance of cross-domain edges between symptoms and within-network connectivity in the unified model potentially reflects that between-network measures capture variance that overlaps with the within-network measures. Simultaneously, EBICglasso’s penalization favors sparser solutions when the parameter space grows, so weak conditional associations have likely shrunk to zero as a consequence. Importantly, the persistence of symptom—DN–SA edges in both the between-only and unified models indicates these are more robust unique associations after accounting for other connectivity measures.

Overall, these findings support that internalizing problems are in part linked to atypical network connectivity of DN regions in youth [71]. However, contrary to previous studies that reported an interacting role of sex onto the relationship between internalizing problem development and DN connectivity [41, 78–80], we did not find evidence for sex differences in the examined symptom-functional connectivity networks. Even though the prevalence and characteristics of internalizing problems vary between boys and girls [2], our results indicate that the associations between symptoms and functional connectivity are similar among sexes in mid adolescence. Given evidence of sex differences in the restructuring and reorganization of functional networks during adolescence [81, 82], longitudinal studies with information on sex and pubertal timing are needed to better understand underlying neural mechanisms of emerging internalizing problems in adolescence [6, 83].

Moreover, our analyses showed that within-network connectivity of the DN at baseline (around age 10) was negatively associated with self-reported feelings of worthlessness measured four years later. This link disappeared after accounting for self-reported worthlessness at the 6-month follow up, reflecting that functional connectivity at age 10 was not associated with a change in worthlessness symptoms between 10.5 and 14. Atypical brain network organization associated with depression can already be detected in youth at age 10 before the onset of depression symptoms [21]. Our results indicate that the relationship between self-reported worthlessness and within-connectivity patterns of DN regions is already present around age 10. Additionally, links between internalizing symptoms and DN-SA connectivity found in 14-year-olds, could potentially reflect the starting encroachment of the SA onto the DN regions that subsequently leads to a nearly twofold expanded SA in adult patients with depression [21]. It should be noted that the timing of specialization varies among functional networks [81, 84], whereby links between internalizing problems and connectivity between SA and FP might emerge in later developmental stages [71]. Our exploratory analyses on longitudinal associations between internalizing symptoms and functional connectivity cannot capture their dynamic interplay. Future studies leveraging additional timepoints from forthcoming data releases may enable more fine-grained longitudinal tracking and modeling of these dynamic processes, thereby improving our understanding of how symptom-brain associations evolve over time.

It has been suggested that previous attempts to identify specific neuroimaging correlates of depression and anxiety have been impeded by high levels of symptom heterogeneity and cross-disorder comorbidity [6, 85, 86]. MDD, for example, is characterized by a large variability in symptom profiles [66, 87]. Nonetheless, there is accumulating evidence that depressive symptom profiles and trajectories are associated with connectivity-based biotypes [78, 88–90], and network-specific dynamics have been found to underlie symptom heterogeneity in young adults with depression [37]. Leveraging comprehensive precision neuroimaging, Lynch and colleagues [21] provided further evidence that functional connectivity changes are associated with symptom-specific changes over time and predict future anhedonia in adult depression. The heterogeneity of internalizing problems might thus be partially reflected in functional connectivity patterns.

Our findings expand on previous research that has linked global measures of internalizing problems to atypical DN, FP, and SA connectivity [24, 26, 91] and provide evidence of symptom-specific links between internalizing symptoms and functional connectivity in youth, i.e. unique associations while exerting stringent statistical control. By contrast, the Pearson correlations between internalizing symptoms and functional connectivity (Fig. 1E) capture bivariate associations without accounting for shared variance with other symptoms or connectivity measures. The observed differences between regularized partial correlations and Pearson correlations therefore underscore the extent of shared variance among both symptoms and connectivity indices. It remains unclear whether functional connectivity patterns reflect a general psychopathology factor, disorder-specific, or symptom-specific characteristics, or a combination of all [5, 92–94], yet partial associations between symptom and functional connectivity measures potentially highlight meaningful symptom-specific characteristics beyond shared neural mechanism of internalizing problems[5, 94, 95]. By integrating brain and symptom networks we demonstrated a novel approach toward exploring symptom-specific associations with functional connectivity, which sidesteps the limitations of rigid categorical diagnostic classifications and offers a new opportunity to identify mechanisms that contribute to the development and persistence of symptom interrelationships. Future studies that incorporate genetic, environmental, neural, and psychological factors in integrated or multi-layered networks can shed light onto the multifactorial nature of mental health problems.

Our findings should be interpreted in the context of some limitations. Two challenges with combining symptom data and functional connectivity into one network are their different time scales and edge weight coefficients. Edge weights in symptom networks typically represent partial correlations, while the seed-based functional connectivity utilizes Pearson’ product moment (full) correlation coefficients to represent relationships between different brain regions [55, 96]. We considered average (z-transformed) functional connectivity within and between DN, FP, and SA as phenotypes of the brain and treated these measures equivalent to symptom ratings. Thus, we cannot infer how internalizing symptoms and individual connections between regions of networks relate. It should also be noted that methodological differences in FC analyses restrict the direct comparison between studies, as brain parcellations determining network borders [15, 97] and further preprocessing steps and statistical approaches vary between studies and might affect their findings [98, 99]. Additionally, small effect sizes of brain-behavior associations imply that cross-domain edges (between symptom and brain nodes) are modest compared to within-domain edge weights and their estimated bootstrapped CIs reflect limited precision of the edge weight estimation [60]. By using a regularization technique, we are reducing the risk of false positives yet simultaneously risk that the edge weights of small existing cross-domain associations are set to zero. Moreover, as the within-domain associations were particularly strong, the cross-domain relationships might have been overly minimized due to the use of conditional associations. The non-normal distribution of clinical data—a common feature in population samples—and the 3-point Likert scale of the BPM represent additional limitations of this study. Although our approach followed recommendations in the literature (e.g., [100, 101]), these choices remain limitations that warrant evaluation using modeling frameworks specifically developed for ordinal and count data [100, 101]. Lastly, the limited number of internalizing symptoms assessed in the Brief Problem Monitor does not reflect the full spectrum of internalizing problems. Key symptoms of depression and anxiety disorders such as anhedonia, sleep disturbances, avoidance behaviors, and somatic complaints are not included in our analysis and thus limit the generalizability of our results to these disorders. Future studies should thus utilize questionnaires that assess a broader set of symptoms that are potentially related to functional [102–105].

## Conclusion

We examined symptom-specific associations between internalizing problems and functional connectivity measures of DN, FP, and SA in a large population-based adolescent sample. By combining self-reported symptom data and functional network connectivity measures into one network, we identified small conditional relationships between internalizing symptoms and connectivity within these three networks and between DN and SA. Next to highlighting that internalizing problems and functional connectivity are linked on a symptom-specific level in youth, our study showcases the value of integrating symptom and neuroimaging data in the same network and adapting a symptom-specific approach.

## Supplementary Information

Below is the link to the electronic supplementary material.

Supplementary file1 (DOCX 985 KB)

## Data Availability

Data from the ABCD Study is available through the National Institutes of Health Data Archive (NDA; https://nda.nih.gov/abcd).
