# Supplementary material for: Symptom-specific links between internalizing problems and functional connectivity in adolescents: a network analysis
Source: Eur Child Adolesc Psychiatry. 2026 Feb 25;35(6):1839–54. doi: 10.1007/s00787-026-02989-6 (PMC13337947; doi:10.1007/s00787-026-02989-6)
Supplement: Supplementary file 1 — Supplementary file1 (DOCX 985 KB) [file 787_2026_2989_MOESM1_ESM.docx]

**Supplementary Material**

**Symptom-Specific Links between Internalizing Problems and Functional Connectivity in Adolescents: A Network Analysis**

Valerie Karl, Ludvig D. Bjørndal, Eira R. Aksnes, Irene J.E. Teulings, Niamh MacSweeney, Dani Beck, Lars T. Westlye, Omid V. Ebrahimi, Christian K. Tamnes

1. Figure S1. Overview of Data Collection Timepoints
2. Figure S2. Default, Frontoparietal, and Salience Networks
3. Figure S3. Within Default Network Connectivity Before and After Harmonization with neuroComBat
4. Figure S4. Within Default Network Connectivity at Age 10 Before and After Harmonization with neuroComBat
5. Table S1. Edge Weight Matrix Within-Network Connectivity
6. Table S2. Edge Weight Matrix Between-Network Connectivity
7. Figure S5. Node Strength of Within- and Between-Connectivity Networks
8. Table S3. Exploratory Analysis: Edge Weight Matrix Baseline Within-Network Connectivity
9. Table S4. Exploratory Analysis: Edge Weight Matrix Baseline Between-Network Connectivity
10. Figure S6. Baseline Sample Characteristics
11. Figure S7. Node Strength of Both Within- and Between-Connectivity in a Unified Network
12. Figure S1


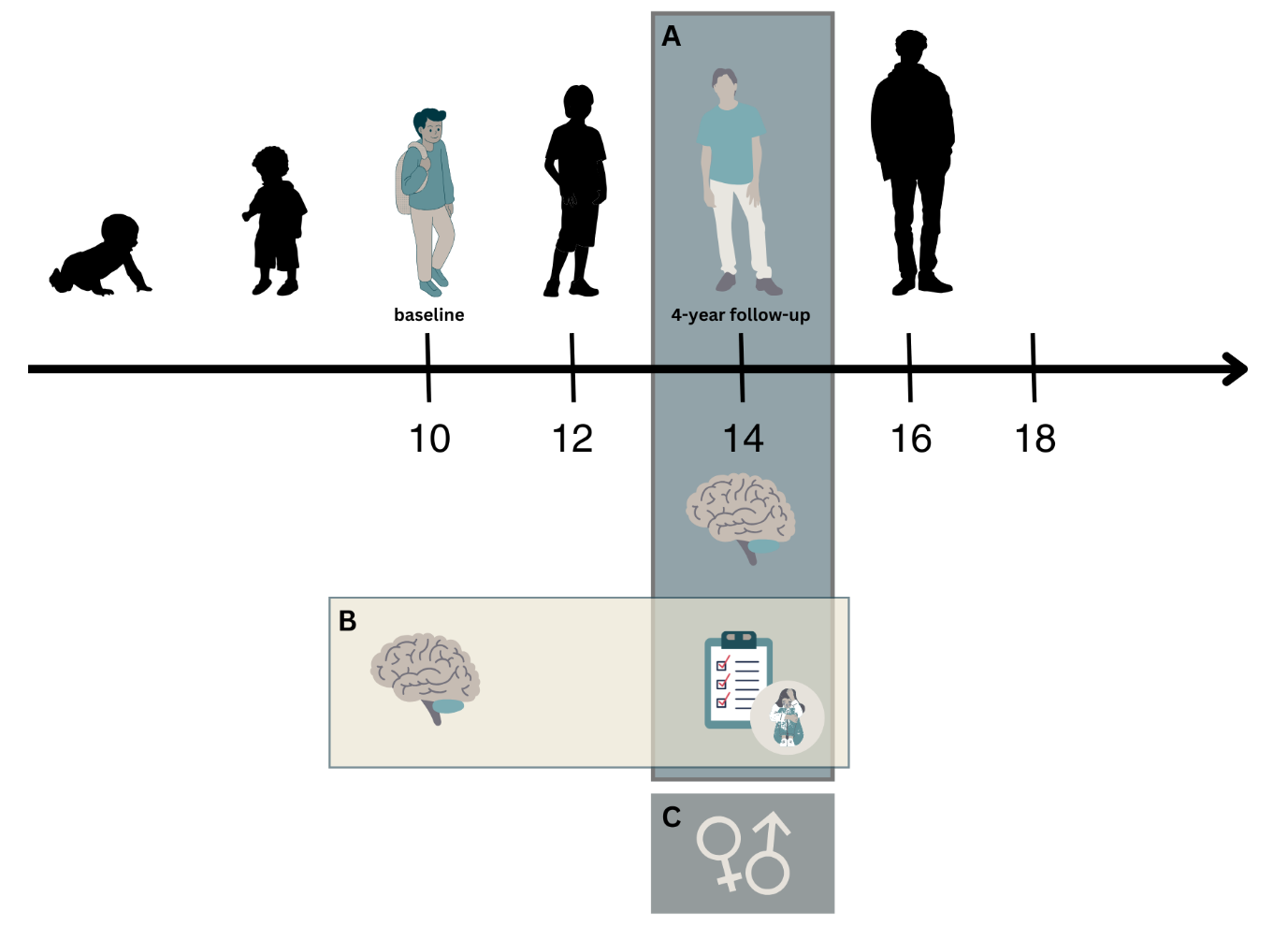


**Figure S1.** *Overview of Data Collection Timepoints.* **A:** The main analysis focused on neuroimaging and mental health data available from the ABCD Study 4-year follow-up timepoint (N = 2426). **B:** To test the association between functional connectivity at age 10 and internalizing problems at age 14, we used neuroimaging data from the baseline timepoint and symptom data from the 4-year follow-up timepoint (N = 1997). **C:** Sex-differences were tested with data from the 4-year follow-up timepoint for participants from the main analysis (N = 2426).

1. Figure S2


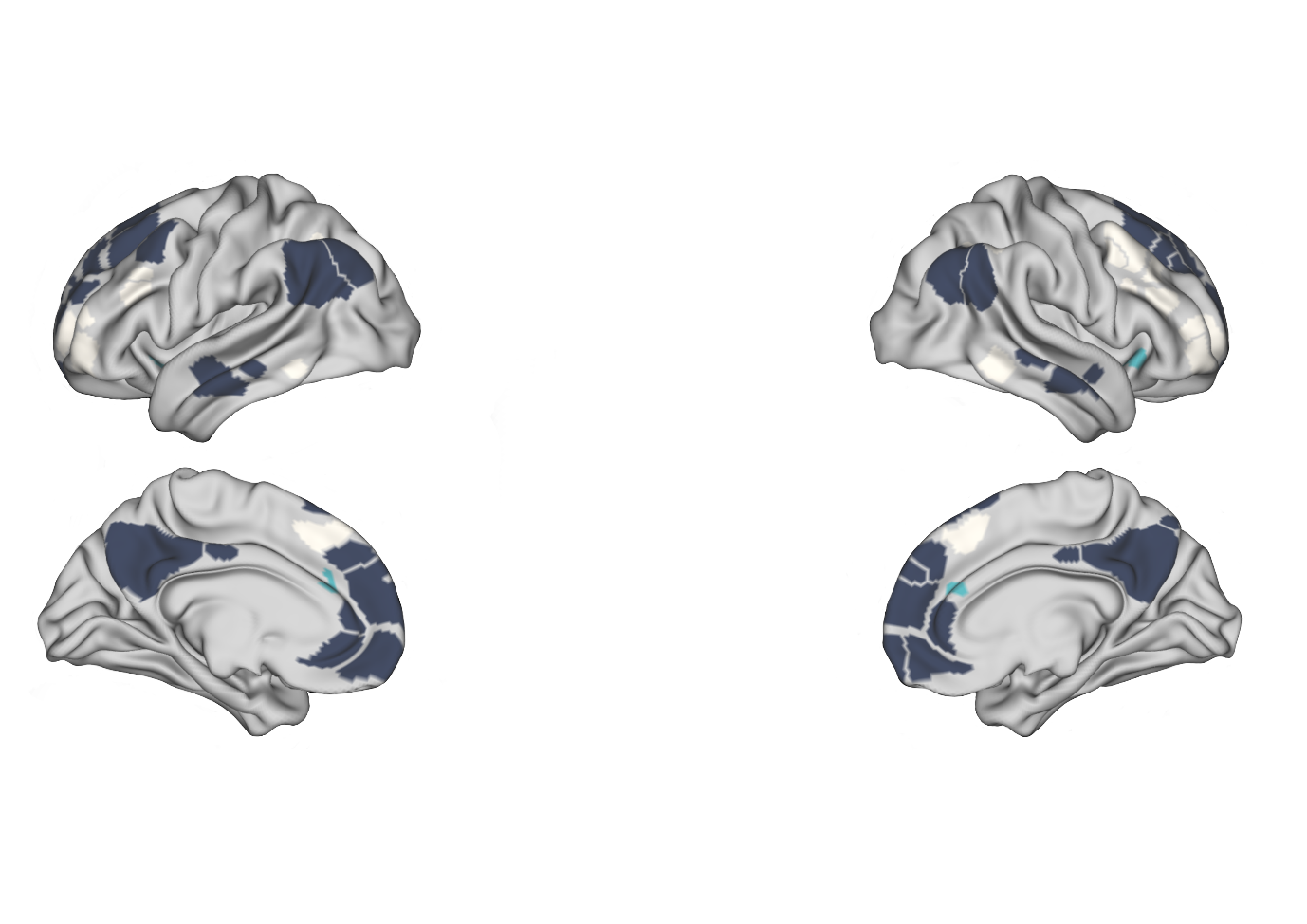
**Figure S2.** *Default, Frontoparietal, and Salience Networks.* Displayed are parcels belonging to the Default (dark blue), Frontoparietal (beige), and Salience (turquoise) Network according to the Gordon parcellation (1).

1. Figure S3


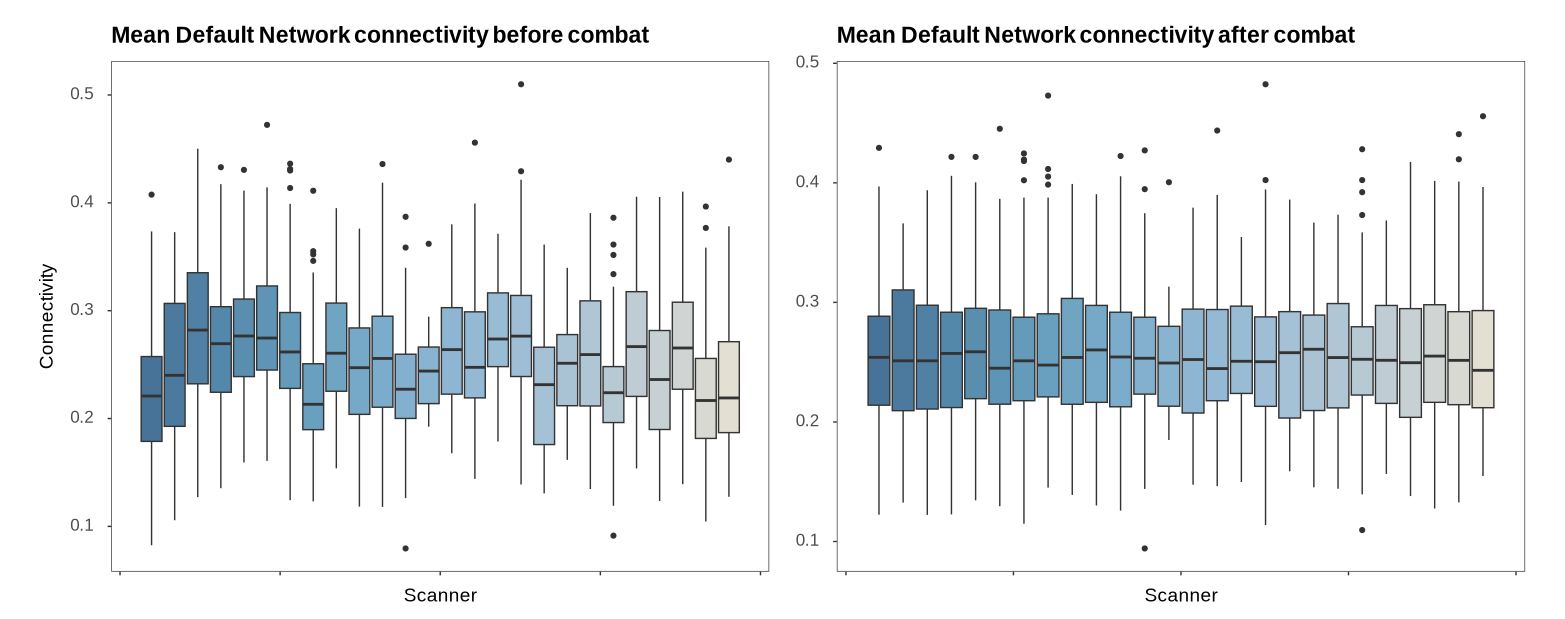


**Figure S3.** *Within Default Network Connectivity Before and After Harmonization with neuroComBat.* Data (collected at age 14) was adjusted for scanner effects using neuroComBat (2) using internalizing symptom items, age, and sex as covariates.

1. Figure S4


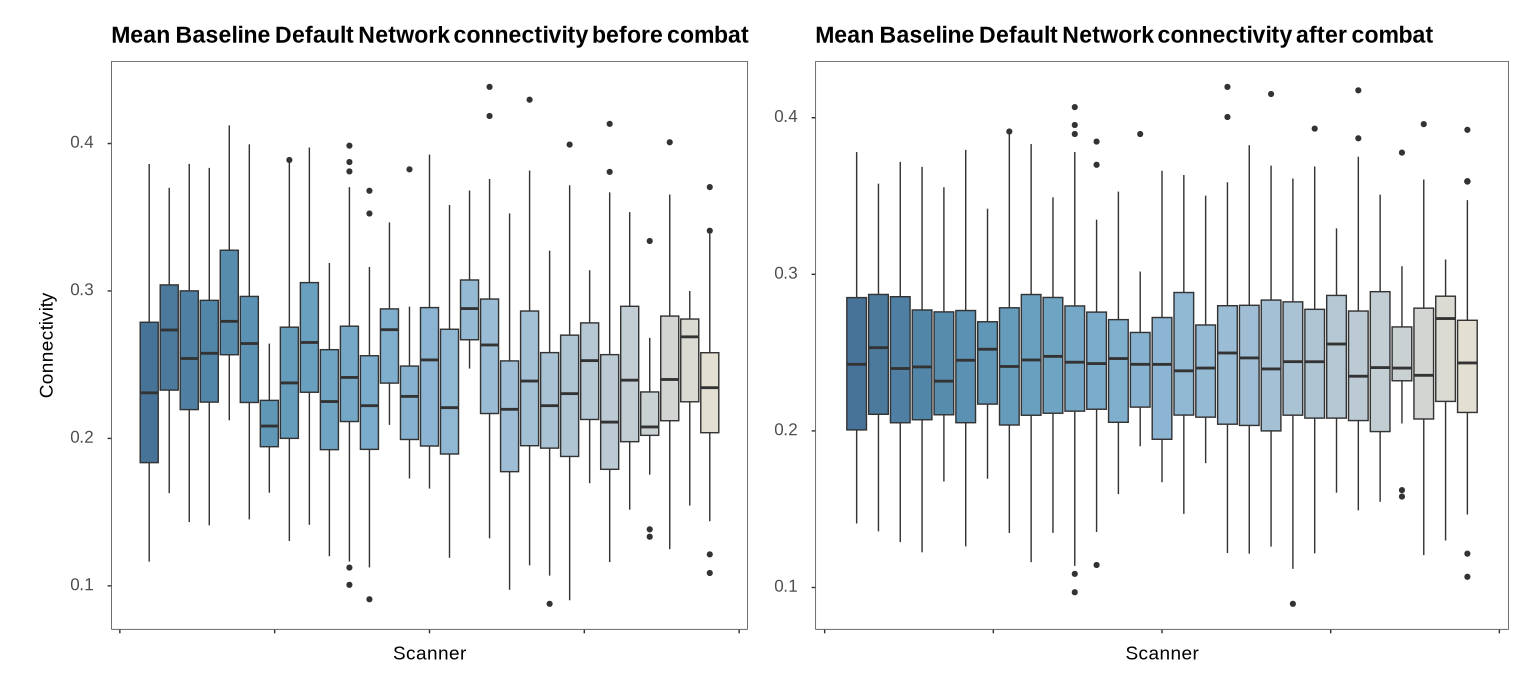


**Figure S4.** *Within Default Network Connectivity at Age 10 Before and After Harmonization with neuroComBat.* Data (collected at age 10, i.e. the ABCD Study baseline timepoint) was adjusted for scanner effects using neuroComBat (2) using internalizing symptom items, age at baseline, and sex as covariates.

1. Table S1: Edge Weight Matrix Within-Network Connectivity

| **Table S1.** *Within-Network Connectivity Edge Weights* | | | | | | | | | |
| --- | --- | --- | --- | --- | --- | --- | --- | --- | --- |
| *Node* | DN | FP | SA | Worth | Fearful | Guilty | Self_con | Sad | Worry |
| DN | **-** | 0.191 | 0.068 | -0.012 | 0.000 | -0.017 | 0.000 | 0.000 | 0.000 |
| FP |  | **-** | 0.090 | 0.000 | 0.009 | 0.000 | 0.000 | 0.000 | 0.000 |
| SA |  |  | **-** | -0.011 | 0.000 | 0.000 | 0.000 | 0.000 | 0.000 |
| Worth |  |  |  | **-** | 0.094 | 0.155 | 0.101 | 0.438 | 0.043 |
| Fearful |  |  |  |  | **-** | 0.195 | 0.195 | 0.060 | 0.368 |
| Guilty |  |  |  |  |  | **-** | 0.116 | 0.101 | 0.102 |
| Self_con |  |  |  |  |  |  | **-** | 0.079 | 0.175 |
| Sad |  |  |  |  |  |  |  | **-** | 0.123 |
| Worry |  |  |  |  |  |  |  |  | - |
| *Note.* DN = Default Network; FP = Frontoparietal Network; SA= Salience Network; Worth = Worthlessness; Self-con = Self-consciousness. | | | | | | | | | |

1. Table S2. Edge Weight Matrix Between-Network Connectivity

| **Table S2.** *Between-Network Connectivity Edge Weights* | | | | | | | | | |
| --- | --- | --- | --- | --- | --- | --- | --- | --- | --- |
| *Node* | DN_FP | DN_SA | FP_SA | Worth | Fearful | Guilty | Self_con | Sad | Worry |
| DN_FP | **-** | 0.189 | 0.131 | 0.000 | 0.000 | 0.000 | 0.000 | 0.000 | 0.000 |
| DN_SA |  | **-** | 0.000 | 0.000 | 0.003 | 0.000 | 0.000 | 0.004 | 0.000 |
| FP_SA |  |  | **-** | 0.000 | 0.000 | 0.000 | 0.000 | 0.000 | 0.000 |
| Worth |  |  |  | **-** | 0.093 | 0.151 | 0.098 | 0.423 | 0.045 |
| Fearful |  |  |  |  | **-** | 0.189 | 0.191 | 0.061 | 0.356 |
| Guilty |  |  |  |  |  | **-** | 0.112 | 0.099 | 0.101 |
| Self_con |  |  |  |  |  |  | **-** | 0.077 | 0.171 |
| Sad |  |  |  |  |  |  |  | **-** | 0.120 |
| Worry |  |  |  |  |  |  |  |  | - |
| *Note.* DN_FP = Connectivity between Default Network and Frontoparietal Network; DN_SA = Connectivity between Default Network and Salience Network; FP_SA= Connectivity between Frontoparietal Network and Salience Network; Worth = Worthlessness; Self-con = Self-consciousness. | | | | | | | | | |

1. Figure S5


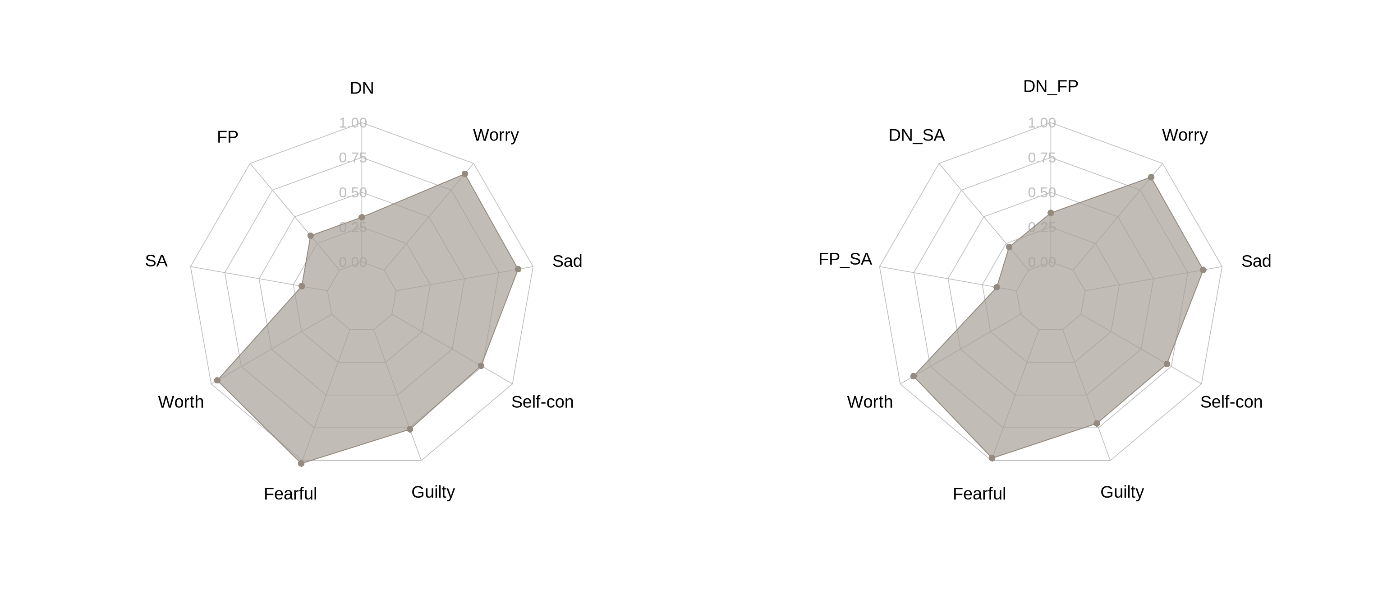


**Figure S5***: Node Strength of Within- and Between-Connectivity Networks.* Node strength for internalizing symptoms and within-network (left) and between-network (right) functional connectivity measures at age 14.

1. Table S3. Exploratory Analysis: Edge Weight Matrices (Baseline Within-Network Connectivity)

| **Table S3.** *Within-Network Connectivity Edge Weights with Baseline Functional Connectivity* | | | | | | | | | |
| --- | --- | --- | --- | --- | --- | --- | --- | --- | --- |
| *Node* | DN | FP | SA | Worth | Fearful | Guilty | Self_con | Sad | Worry |
| DN | **-** | 0.190 | 0.106 | -0.010 | 0.000 | 0.000 | 0.000 | 0.000 | 0.000 |
| FP |  | **-** | 0.038 | 0.000 | 0.000 | 0.000 | 0.000 | 0.000 | 0.000 |
| SA |  |  | **-** | 0.000 | 0.000 | 0.000 | 0.000 | 0.000 | 0.000 |
| Worth |  |  |  | **-** | 0.102 | 0.155 | 0.097 | 0.439 | 0.048 |
| Fearful |  |  |  |  | **-** | 0.204 | 0.197 | 0.043 | 0.377 |
| Guilty |  |  |  |  |  | **-** | 0.111 | 0.080 | 0.091 |
| Self_con |  |  |  |  |  |  | **-** | 0.083 | 0.159 |
| Sad |  |  |  |  |  |  |  | **-** | 0.139 |
| Worry |  |  |  |  |  |  |  |  | - |
| *Note.* Functional Connectivity was collected at the baseline ABCD Study timepoint (at age 10) and internalizing symptom data at age 14. DN = Default Network; FP = Frontoparietal Network; SA= Salience Network; Self-con = Self-consciousness. | | | | | | | | | |

1. Table S4. Exploratory Analysis: Edge Weight Matrices (Baseline Between-Network Connectivity)

| **Table S4.** *Between-Network Connectivity Edge Weights with Baseline Functional Connectivity* | | | | | | | | | |
| --- | --- | --- | --- | --- | --- | --- | --- | --- | --- |
| *Node* | DN_FP | DN_SA | FP_SA | Worth | Fearful | Guilty | Self_con | Sad | Worry |
| DN_FP | - | 0.214 | 0.155 | -0.000 | -0.000 | -0.000 | -0.000 | -0.000 | -0.000 |
| DN_SA |  | - | 0.000 | -0.000 | -0.000 | -0.000 | -0.000 | -0.000 | -0.000 |
| FP_SA |  |  | - | -0.000 | -0.000 | -0.000 | -0.000 | -0.000 | -0.000 |
| Worth |  |  |  | - | 0.102 | 0.155 | 0.097 | 0.439 | 0.048 |
| Fearful |  |  |  |  | - | 0.204 | 0.197 | 0.043 | 0.377 |
| Guilty |  |  |  |  |  | - | 0.111 | 0.080 | 0.091 |
| Self_con |  |  |  |  |  |  | - | 0.083 | 0.159 |
| Sad |  |  |  |  |  |  |  | - | 0.139 |
| Worry |  |  |  |  |  |  |  |  | - |
| *Note.* Functional connectivity was obtained from the ABCD Study baseline timepoint four years prior to the report of internalizing symptoms at age 14. DN_FP = Connectivity between Default Network and Frontoparietal Network; DN_SA = Connectivity between Default Network and Salience Network; FP_SA= Connectivity between Frontoparietal Network and Salience Network; Self-con = Self-consciousness. | | | | | | | | | |

1. Figure S6


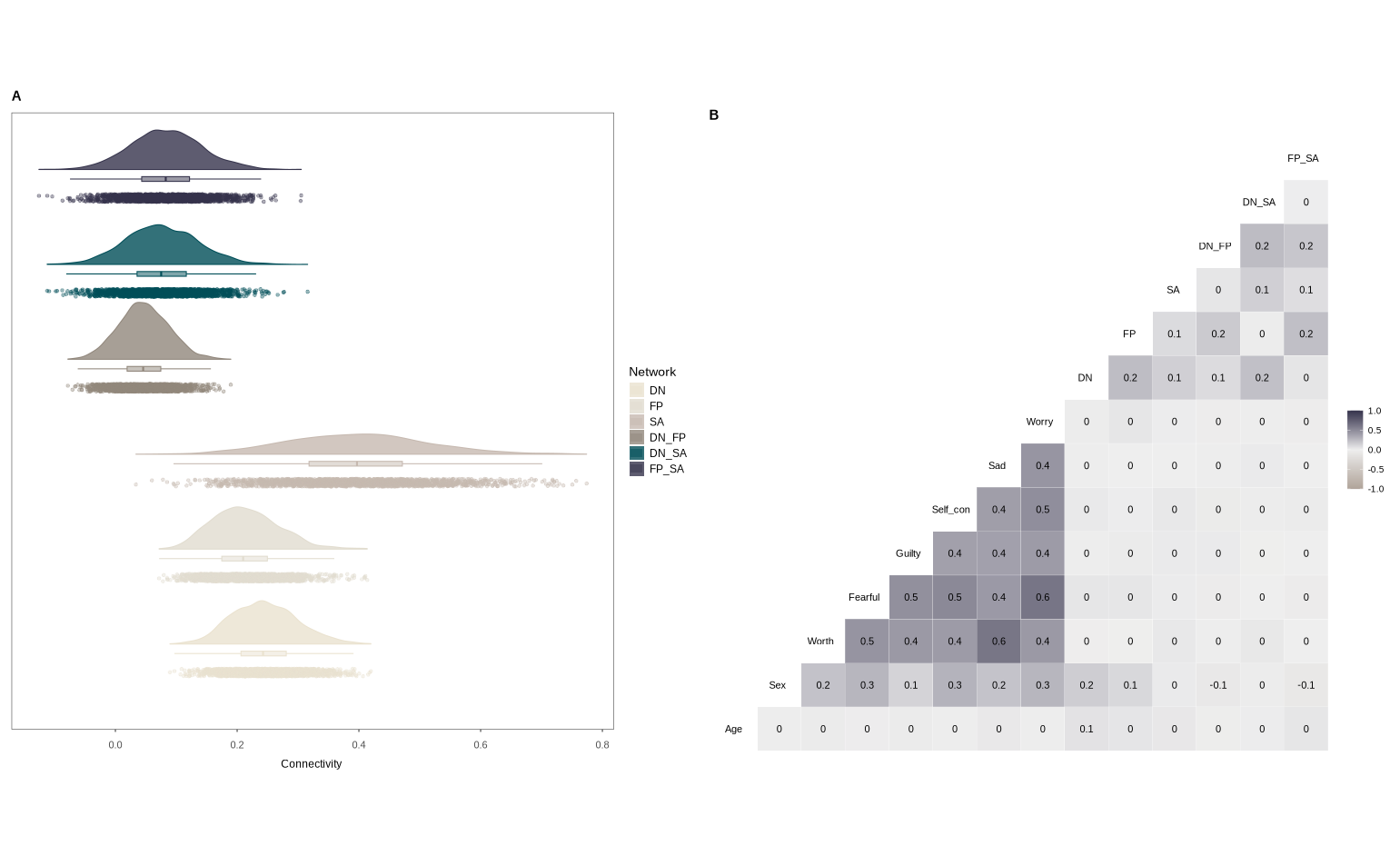


**Figure S6.** *Baseline Sample Characteristics. A:* Average network connectivity strength with data collected at age 10, i.e. the ABCD Study baseline timepoint. B: Pearson’s correlation coefficients between functional connectivity, symptom ratings, age and sex. Age and functional connectivity were observed at age 10.

1. Figure S7


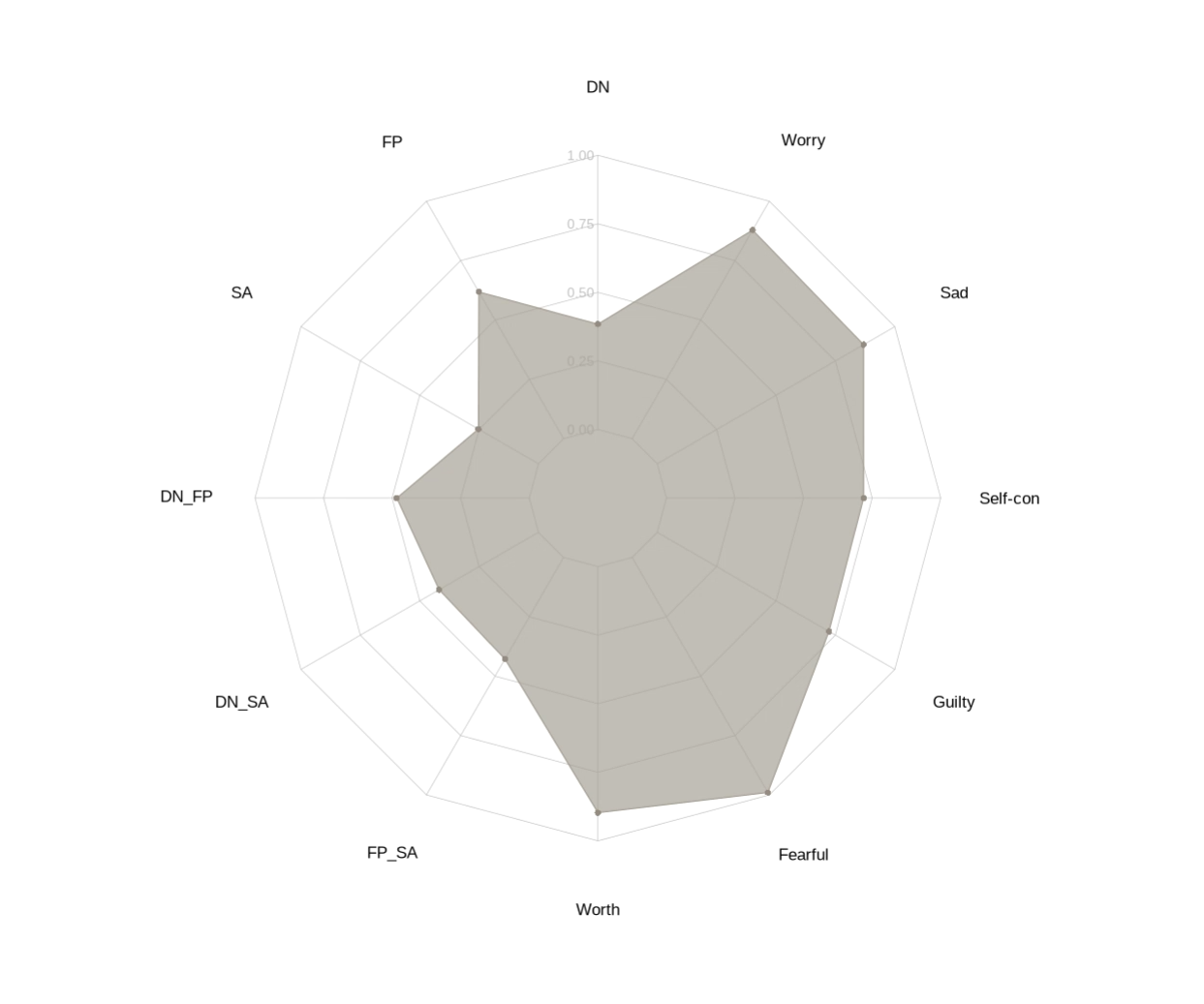


**Figure S7***: Node Strength of Both Within- and Between-Connectivity in a Unified Network.* Node strength for internalizing symptoms and within-network and between-network functional connectivity measures at age 14.

**References**

1. Gordon EM, Laumann TO, Adeyemo B, Huckins JF, Kelley WM, Petersen SE. Generation and Evaluation of a Cortical Area Parcellation from Resting-State Correlations. Cereb Cortex. 2016 Jan;26(1):288–303.

2. Fortin JP. neuroCombat: Harmonization of multi-site imaging data with ComBat. 2021;
